# Biological Barrier Models-on-Chips: A Novel Tool for Disease Research and Drug Discovery

**DOI:** 10.3390/bios15060338

**Published:** 2025-05-26

**Authors:** Giusi Caragnano, Anna Grazia Monteduro, Silvia Rizzato, Gianluigi Giannelli, Giuseppe Maruccio

**Affiliations:** 1 Omnics Research Group, Department of Mathematics and Physics “Ennio De Giorgi”, Institute of Nanotechnology, CNR—Nanotec and INFN Sezione di Lecce, University of Salento, Via per Monteroni, 73100 Lecce, Italy; giusi.caragnano@unisalento.it (G.C.); annagrazia.monteduro@unisalento.it (A.G.M.); silvia.rizzato@unisalento.it (S.R.); 2 National Institute of Gastroenterology “Saverio de Bellis”, IRCCS Hospital, Castellana Grotte, 70013 Bari, Italy; gianluigi.giannelli@irccsdebellis.it

**Keywords:** organ-on-chip, biological barrier, microfluidic platforms, personalized medicine, biosensors

## Abstract

The development of alternatives to animal models and traditional cell cultures has led to the emergence of organ-on-chip (OoC) systems, which replicate organ functions under both physiological and pathological conditions. These microfluidic platforms simulate key tissue interfaces—such as tissue–air, tissue–liquid, and tissue–tissue interactions—while incorporating biomechanical stimuli to closely resemble in vivo environments. This makes OoC systems particularly suitable for modeling biological barriers such as the skin, the placenta, and the blood–brain barrier, which play essential roles in maintaining homeostasis. This review explores various biological barrier models that can be replicated using the OoC technology, discussing the integration of induced pluripotent stem cells (iPSCs) to advance personalized medicine. Additionally, we examine the methods for assessing barrier formation, including real-time monitoring through integrated sensors, and discuss the advantages and challenges associated with these technologies. The potential of OoC systems in disease modeling, drug discovery, and personalized therapeutic strategies is also highlighted.

## 1. Introduction

Biological barriers have a critical protective and regulatory role in the human body, separating internal compartments from the outer environment or external stimuli and regulating the passage of ions, biomolecules, pathogens, and drugs. Their dysfunction can lead to various diseases, and for this reason, they have been largely investigated [1,2,3,4]. However, traditional models present relevant limitations. On one hand, animal studies [5] are limited by interspecies differences and ethical concerns. On the other, 2D cell cultures fail in reproducing the full complexity of in vivo barrier physiology due to a lack in cellular diversity, architecture, and mechanical cues. For instance, Chen et al. demonstrated the importance of palmitoylation enzyme activity in maintaining skin barrier integrity through animal studies. The researchers used knock-in mice carrying a DQ-to-AA ZDHHC13 mutation, which renders the enzyme inactive, and this caused a phenotype similar to the knock-out model, with hair loss and skin inflammation. Then, they identified possible proteins regulated by ZDHHC13 using quantitative proteomics, and three key substrates were identified, including loricrin and peptidyl arginine deiminase type III, whose palmitoylation proved critical for the stability of these proteins in vivo [6]. Similarly, Li et al. used zebrafish to investigate nanoparticle transport across biological barriers, including the BBB, BRB, and gastrointestinal barrier. In particular, coumarin-6 nanocrystals (C6-NCs) having sizes of 70 and 200 nm, fabricated by the anti-solvent precipitation method, and characterized with Malvern’s Nano-Zetasizer were used to measure dynamic light scattering. The transport of these nanocrystals across a number of biological barriers including the BBB, gastrointestinal barrier, and chorion was tested at different developmental stages of zebrafish, including embryos, larvae, and adults, and it was observed that the nanoparticles of smaller size in adults were transported more efficiently through lipid rafts and exhibited greater uptake at the cellular level [7]. However, these conventional methods present limitations such as ethical concerns, poor translatability of the results to human physiology, and lack of cellular complexity.

Organ-on-chip (OoC) technology has recently emerged as a promising alternative to address these limitations [8]. These microfluidic devices are designed to reproduce organ functions and facilitate disease modeling. They provide a more accurate platform than traditional systems for studying diseases and developing new therapies within a controlled microenvironment by replicating in vivo conditions, multicellular interactions, tissue interfaces, and complex functionalities of barriers/organs, incorporating biomechanical forces (like shear stress and cyclic strain) and allowing for co-culturing relevant cell types [9,10,11,12,13]. OoC models permit the study of the integrity of cell junctions and barriers as a function of disease progression and drug administration. Chemical and physical gradients can be created through a laminar flow in order to investigate their influence [12,14,15,16,17,18]. The use of induced pluripotent stem cells (iPSCs) allows for retaining the genetic characteristics of the donor by differentiating them into the cell types relevant for the target model, including biological barriers [19,20,21,22,23]. Furthermore, the integration of miniaturized biosensors in OoC platforms enables the real-time monitoring of physiological parameters like pH, oxygen concentration, glucose consumption, as well as transport mechanisms [15], barrier integrity, and cellular activities [15,24,25,26]. The integration of sensors also facilitates drug discovery by providing immediate feedback on the efficacy of pharmaceutical interventions.

They surpass animal models by eliminating ethical concerns, improving translatability between species and reducing the variability in drug response. While compared to 2D cultures, OoC systems solve the lack of cellular heterogeneity and compartmentalization through the use of cell co-cultures and microchannels separated by porous membranes or pillars. In fact, the presence of compartments can increase the control of the microenvironment by confining the cells, and the presence of interfaces can help to imitate the structural divisions present at the organ level and allow for studies of transcellular transport, absorption, and secretion. At the same time, they improve 3D cultures by introducing biomechanical forces such as shear stress and cyclic strain and flow, which play a fundamental role in tissue differentiation and function. In these microfluidic platforms, fluid control has proven to be of great help, being essential for the diffusion of molecules in cells, the supply of nutrients, the elimination of waste, and cellular polarity. Furthermore, since the flow is laminar because the diameter of the microfluidic channel is less than one millimeter, it allows for the formation of chemical [14] and physical gradients useful for the study of the integrity of cell junctions, microbial growth, and directional cell migration [12,15,16,17,18]. Additionally, OoCs can integrate biosensors for real-time cellular activity monitoring, enhancing their potential for drug discovery and personalized medicine. Most sensors integrated into OoC platforms feature a detection element to which analytes are bound, a component that transduces the binding events into output signals, and a device that converts the output signal into appropriate readings [15,24]. Optical and electrochemical sensors are the most popular ones because of their ability to adapt to small chip sizes and their sensitivity [15]. Electrochemical sensors may be useful within a gut-on-chip, where intestinal cells are subjected to different concentrations of oxygen depending on whether they make up the lumen or the intestinal mucosa. These sensors can also measure changes in pH and temperature or analyze metabolic parameters such as glucose consumption [15]. As we continued examining the possibilities of using biosensors in organ-on-chip systems, we also discovered multiple-electrode arrays (MEAs) and transepithelial electrical resistance sensors that can be used to record cardiac pulsations [15,25] or to measure the integrity of the intestinal barrier [26] and even mechanical sensors to simulate blood flow pressure or to monitor the stiffness of engineered tissues on chips [24].

Table 1 provides a comparison of different study models for investigating diseases related to biological barriers [9,10,11,13,15,16,17,24,27,28,29,30,31]. Among the various applications of organs-on-chips are the creation of disease models that reproduce the pathological conditions of organs, offering a better understanding of the mechanisms underlying diseases; pharmacological experimentation, which reduces the need for animal testing and improves the prediction of how drugs might behave in the human body; and finally the possibility of creating personalized platforms to study targeted therapies, improving the effectiveness of treatments for individuals [12].

In fact, one of the major challenges in drug development is the variability in drug response among individuals of the same species due to the presence of single-nucleotide polymorphisms (SNPs), which makes it difficult to identify the most suitable treatment and minimize the side effects. Through genome-wide association studies, hundreds of genetic variants related to genetic diseases and drug efficacy have been identified, allowing groups of individuals to be stratified according to their response to a certain treatment or the development of a certain disease [32].

A promising approach to overcoming this challenge is personalized therapy, particularly through the use of induced pluripotent stem cells (iPSCs). iPSCs are derived from skin biopsies or blood samples and can be reprogrammed into stem cells that retain the donor’s genetic characteristics and can differentiate into any cell type in the human body [19,20,21]. The combination of iPSC technology with organ-on-chip platforms has led to the development of innovative tools that have ethical advantages over those that use embryonic stem cells (ESCs) [22], such as the placenta-on-chip, with iPSC-derived trophoblasts, and the blood–brain barrier-on-chip, achieved by differentiating iPSCs into brain microvascular endothelial cells (iBMECs) [23]. This emerging technology could revolutionize personalized medicine, enabling the testing of patient-specific drugs and the optimization of therapeutic strategies for each individual.

**Figure 1:**
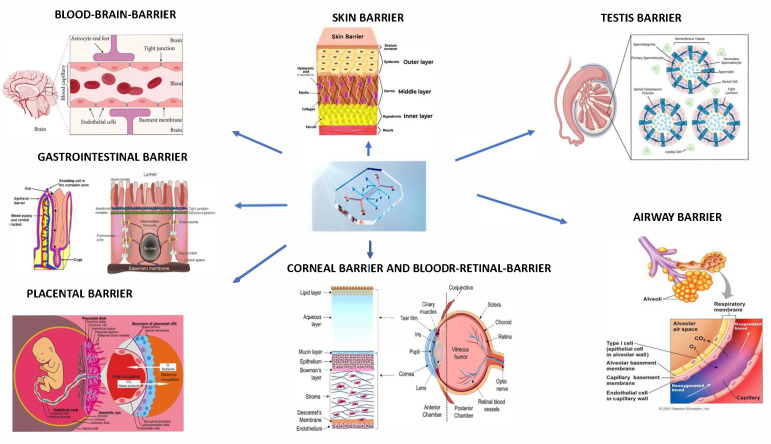
Schematic overview of the various biological barriers on chips described in this work.

In the following sections, we will discuss various organ-on-chip models used to replicate biological barriers (shown in Figure 1) such as the blood–brain barrier, the skin, the placenta, and the gastrointestinal barrier, exploiting also iPSC technology. One of the objectives of this study is to provide an overview of the various biological barriers on chips present in the literature, highlighting their advantages over traditional models and also the possible limitations of these technologies(as described in Table 2). Furthermore, this study could be significant as it addresses one of the main challenges of biomedical research, i.e., faithfully reproducing human physiology to improve the predictivity of preclinical studies. This approach has enormous implications in the search for new drugs, in understanding diseases, and in reducing animal experimentation, bringing us ever closer to personalized and more effective medicine by advancing biomedical research and therapeutic development [23,32,33]

## 2. Blood–Brain Barrier (BBB)-On-Chip

The blood–brain barrier (BBB) is a critical component of the central nervous system, separating the brain from the bloodstream and regulating the selective passage of substances through tight junctions. In this way, the BBB performs a protective action against pathogens and harmful substances while maintaining brain homeostasis [34,35,36] but also represents a relevant barrier for drug delivery.

Another crucial barrier within the brain is the blood–cerebrospinal fluid (CSF) barrier, located in the choroid plexuses. This barrier consists of choroid capillaries and choroid epithelial cells, which also possess tight junctions that restrict the permeability between blood and CSF [37,38,39,40]. However, while essential for brain protection, the BBB also complicates drug delivery for treating neurodegenerative diseases such as Alzheimer’s and Parkinson’s diseases, which are becoming more prevalent due to the increased life expectancy. To overcome this limitation, an increasing number of approaches have been developed, such as liposomes, nanoparticles, and polymeric drug delivery systems [40].

To study transport across the BBB, researchers have developed BBB-on-chip models, which have two compartments, one representing the blood, and the other representing the brain tissue. Various designs of BBB-on-chip have been proposed, e.g., the two compartments can be arranged (i) vertically, separated by a porous membrane [41], or (ii) horizontally, separated by hydrogel polymeric scaffolds; in addition, there are models that present artificial hollow tubes in a hydrogel [42]. These platforms can integrate sensors to monitor in real time the presence of molecules and external substances that influence the physiological, metabolic, and pathological functions of the BBB. Among the various sensing technologies used in BBB-on-chip platforms, integrated electrical sensors—particularly those used to measure transendothelial electrical resistance (TEER)—are of particular relevance, as they provide real-time information on the integrity and permeability of the endothelial barrier. In addition to these, optical sensors—both integrated and modular—are commonly employed. These include systems based on surface plasmon resonance (SPR) and optical interferometry, which allow for the label-free detection of molecular interactions and barrier function. Electrochemical sensors also play a significant role by enabling the sensitive and specific detection of target analytes [43,44,45,46,47].

Physiologically relevant data were obtained through TEER measurements on a BBB-on-chip model consisting of human induced pluripotent stem cell (hiPSC)-derived endothelial cells (ECs) in co-culture with astrocytes and pericytes, which were subjected to high shear stress caused by blood flow on the endothelial cells [37,38].

An interesting contribution in the field of BBB-on-chip was made by Tae-Eun Park and collaborators, who created a two-layer microfluidic platform, with the layers separated by a porous membrane, consisting of primary human pericytes and astrocytes and human brain microvascular endothelium derived from induced pluripotent stem cells (iPS-BMVECs), which expresses some of the main characteristics of the BBB, such as the presence of tight junctions and functional efflux pumps. Furthermore, compared to other BBB-on-chip models, it was shown to have increased barrier functionality, acting on the differentiation of BMVECs in hypoxic conditions, as occurs at the beginning of brain formation, when the circulatory system is not yet developed. This device is a potential tool for better drug screening or disease modelling, as it reproduces a microenvironment very similar to that present in vivo [48].

**Figure 2:**
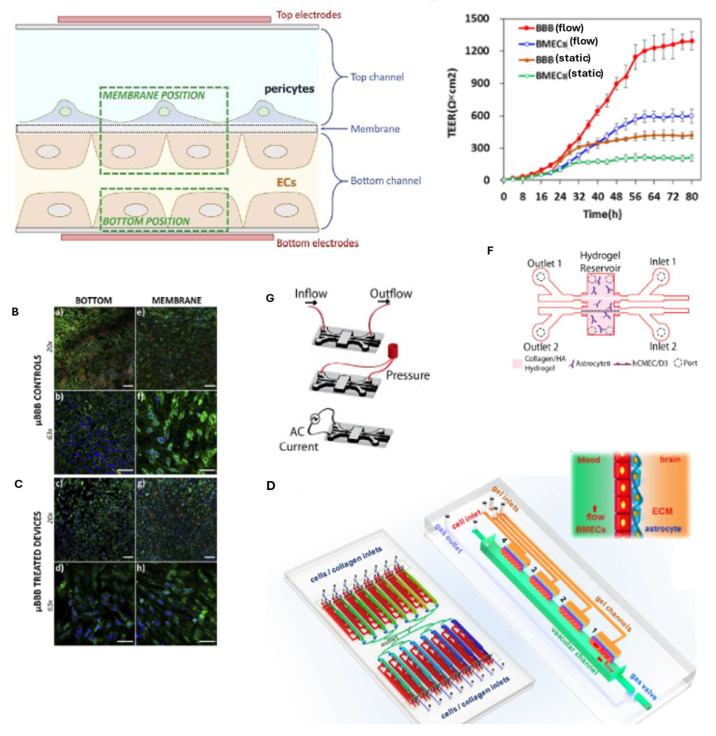
(**A**) Schematic representation of the horizontal section of the BBB-on-chip [49]. (**B**,**C**) Confocal microscopy images of untreated (**a**,**b**,**e**,**f**) and D-mannitol-treated (**c**,**d**,**g**,**h**) BBB after 24 h. Immunochemical staining was performed on the membrane, showing pericytes and endothelial cells (**e**–**h**) and endothelial cells alone (**a**–**d**). Nuclei are stained in blue, ZO-1 tight junctions are stained in green, and VE (vascular endothelial)–cadherin adherens junctions are stained in red. Scale bars: 100 µm for 20× images and 50 µm for 63× images [49]. (**D**) Chip design by Xu et al. [50]. (**E**) TEER measurements of barrier function in the BBB group and brain microvascular endothelial cells (BMECs) under static and flow conditions [50]. (**F**) 3D BBB model by Partyka et al. [51]. (**G**) Representation of fluid flow, cyclic deformation, and TEER measurements in the BBB-on-chip [51].

More recently, Badiola-Mateos et al. [49] monitored the BBB’s integrity dynamically (Figure 2A) on a chip through TEER measurements using integrated microelectrodes and with the support of machine learning techniques. Their model consisted of cyclo-olefin polymer (COP) layers with embedded polycarbonate membranes. Human brain microvascular endothelial cells (hCMEC/D3) were cultured on the lower layer, while bovine pericytes were seeded on the upper layer. After using D-mannitol to induce barrier disruption, the recovery of barrier integrity was monitored via TEER measurements and in real time using interdigitated electrodes on both channels to measure trans-endothelial electrical resistance (TEER), coupled with machine learning techniques, thus introducing a new element compared to the following described works. Immunochemical staining further validated the data (Figure 2B,C), demonstrating the potential of this approach for identifying BBB regeneration in the device and optimizing drug treatments for neurodegenerative disorders.

The functional responses of the BBB under both normal and pathological conditions were investigated by Xu et al. for brain metastasis and glioblastoma. Their model is composed of 16 independent functional units connected by a network of microchannels, each of which consists of a channel for the BBB, a vascular channel, a gas channel, a gas valve, and four gel channels, as shown in Figure 2D. A co-culture of endothelial cells and astrocytes was introduced into the chip within a three-dimensional extracellular matrix, and to characterize the integrity of the barrier, transendothelial electrical resistance (TEER) was measured. In Figure 2E, it can be seen that the presence of flow increases TEER in both brain microvascular endothelial cells (BMECs) and the compartment where there are both BMECs and astrocytes (BBB group). This result highlights the importance of a mechanical stimulus in achieving a more selective vascular compartment. It is known that in animal models, some tumors, such as lung cancer, breast cancer, and melanoma, have a greater propensity to metastasize in the brain rather than in other organs. Using this system, the researchers demonstrated how some tumor cells interact with the BBB. Specifically, they observed that lung, breast, and melanoma cancer cells successfully crossed the BBB, thus obtaining the same results as in animal models, whereas liver cancer cells did not. It is also interesting to note that, despite its aggressiveness, glioblastoma was unable to penetrate the BBB and metastasize into the surrounding vasculature. This finding suggests that BBB-on-chip models can provide valuable insights into tumor progression and potential therapeutic interventions [50].

The impact of mechanical stimuli on the formation of BBB tight junctions was investigated on chip by Partyka et al. using a BBB-on-chip model with two compartments connected by a hydrogel reservoir containing type I collagen, hyaluronan, hCMEC/D3 cells, and astrocytes (Figure 2F). The cells were subjected to fluid flow and cyclic deformation induced by hydrostatic pressure, as shown in Figure 2G. TEER measurements and dextran perfusion assays confirmed that mechanical stimuli facilitated tight junction formation, both in the presence and in the absence of astrocytes in the extracellular matrix [51]. However, the TEER readings are not always reliable due to the difficulty of electrode integration, the presence of air bubbles in the microchannels, or a non-uniform electric field along the culture area [37,38].

These BBB-on-chip advancements highlight the potential of this tool for disease modeling and therapeutic optimization, providing critical insights into drug permeability, neurodegenerative diseases, and brain tumors and paving the way for improved treatments and precision medicine applications. However, there is a pressing need for biosensors capable of detecting molecules with different molecular weights and electrical charges, such as labeled dextrans, rhodamine dyes, antibodies, or albumin. Looking ahead, future advancements in BBB-on-chip platforms are expected to incorporate more sophisticated modular and integrated sensors, providing a complex data stream that would greatly benefit from the use of artificial intelligence (AI) for data analysis and interpretation [43,44,45,46,47].

## 3. Blood–Retinal Barrier (BRB)-on-Chip

**Figure 3:**
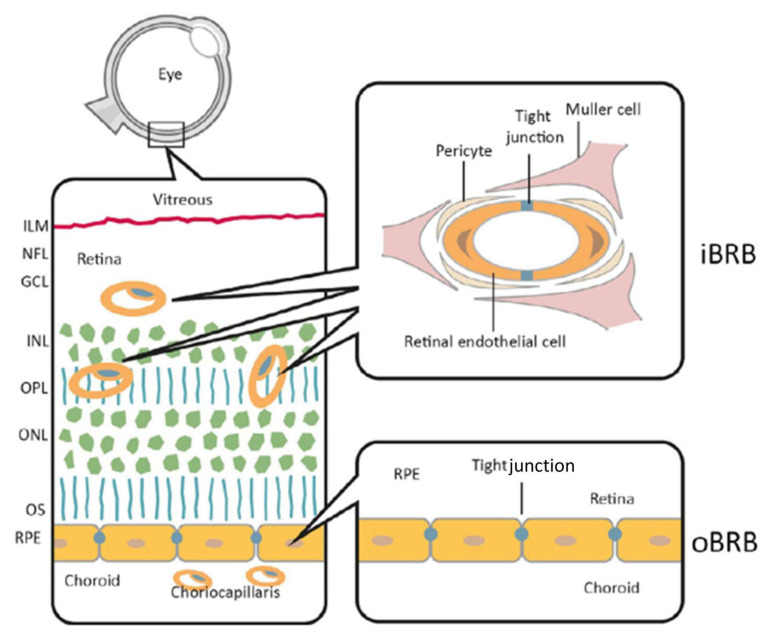
Schematic representation of the inner and outer blood–retinal barrier components. ILM: inner limiting barrier, GCL: ganglion cell layer, INL: outer nuclear layer, OS: outer segments [49].

The blood–retinal barrier (BRB) is essential for preserving the internal environment of the retina by regulating the molecular exchange between the bloodstream and retinal tissues. The BRB consists of two components (Figure 3): the inner BRB (iBRB), formed by retinal vascular endothelial cells coating the vessels of the internal retina and regulating the transport of solutes/molecules from the vascular circulation, and the outer BRB (oBRB), composed of retinal pigment epithelial (RPE) cells and controlling the passage of substances from the retina to the choroid. These barriers prevent harmful substances from entering the retina, while allowing for the passage of essential nutrients [49].

BRB dysfunction is implicated in several ocular diseases, including age-related macular degeneration (AMD) and diabetic retinopathy, both of which can lead to vision impairment. Traditional in vitro models, such as 2D cell cultures, fail to recapitulate the complexity of the BRB, while animal models present ethical and translational challenges. Organ-on-chip technology offers a promising alternative for studying BRB in a physiologically relevant environment, replicating the epithelial phenotype and barrier function [52,53].

Among the proposed BRB-on-chip layouts, one consists of two layers of PDMS with two parallel channels separated by a membrane. These channels contain different cell types, such as human umbilical vein endothelial cells (HUVECs) and retinal pigment epithelium cells (ARPE-19), which are used to simulate choroidal neovascularization (CNV) and investigate angiogenesis.

Another design features a planar model with channels arranged on a single plane. Fibroblasts are seeded in the lateral channels to stabilize the epithelium, while ARPE-19 cells and HUVECs are seeded in the central channels, which are separated from the lateral channels by a layer of 3D fibrin matrix. This system imitates the pathological morphology of CNV and is useful for testing antiangiogenic drugs [52].

**Figure 4:**
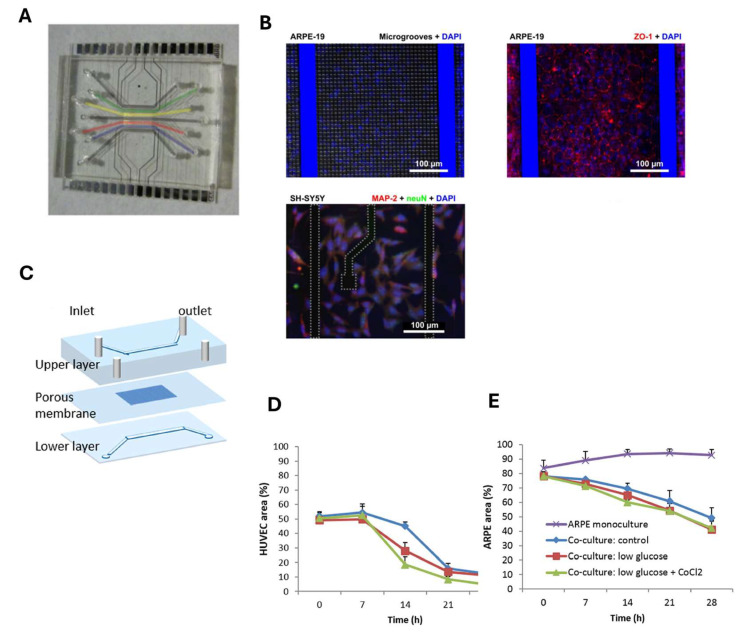
(**A**) Photograph of the microfluidic device described by Yeste et al. [54]. (**B**) Three immunofluorescence images from the experiments carried out by Yeste et al. [54]. (**C**) Schematic representation of the microfluidic device used by Chen et al. [56]. (**D**,**E**) Graphs in which the growth area was quantified at different time points in a control situation, in the presence of low glucose concentrations in the culture medium, and after the addition of CoCl_2_ to enhance hypoxia for the two cell types cultured on the chip [56].

Another more advanced oBRB model was developed by Yaste et al. using a compartmentalized chip that involved the co-culture of primary human retinal endothelial cells (HRECs) to simulate the iBRB and a human neuroblastoma cell line (SH-SY5Y) with an RPE cell line (ARPE-19) to simulate the oBRB. The platform (Figure 4A) consists of a PDMS layer with seven channels, with the first and last channels having no cells, while SH-SY5Y cells are seeded in the three central channels, and ARPE-19 cells and HRECs in the two lateral ones. The PDMS layer, made by photolithography and replica molding techniques, is on a glass layer that has electrodes for TEER readings for the real-time monitoring of occluding junctions, made by lithography and metal deposition, and microgrooves, made by a deep reactive-ion etching (DRIE) process. Figure 4B shows three immunofluorescence images from experiments conducted by Yeste et al. [54]. The top left image displays the ARPE-19 cellular monolayer forming the retinal epithelial barrier within the microchannels of the device, with cell nuclei stained in blue (DAPI). The second image (top-right) shows the distribution of the ZO-1 protein (red), demonstrating the formation of tight junctions, in conjunction with the results from permeability tests and TEER readings, indicating the barrier’s integrity. Both top images were captured using confocal microscopy at 10× magnification. The bottom-left image shows SH-SY5Y cells inside the microfluidic platform, with the nuclei stained in blue and neuronal markers in green (neuN) and red (Map-2). The novelty of this chip lies in the unique placement of the electrodes on the substrate, offering more flexibility and enabling the continuous monitoring of the barrier without interference. Immunofluorescence and permeability assays confirmed the formation of both endothelial and epithelial barriers, highlighting the system’s potential for investigating multi-barrier interactions and retinal disease mechanisms [54].

In a more recent study, Maurissen et al. developed an iBRB-on-chip to study the mechanisms underlying diabetic retinopathy, a microvascular disorder characterized by irreversible retinal loss and rupture of the iBRB. The current animal and in vitro models are not predictive enough to identify new therapeutic targets due to their poor transferability and lack of human relevance. To overcome these limitations, the team recreated an iBRB-on-chip exhibiting the pathological phenotype of diabetic retinopathy. A novelty of this work is the formation of microvascular networks (MVNs) consisting of a tri-culture of primary human retinal microvascular endothelial cells (HRMVECs), primary human retinal microvascular pericytes (HRPs), and primary human retinal astrocytes (HRAs) in a ratio of 1:1:1 (as in the human retina) in a fibrin gel, as each of these three cell types undergoes pathophysiological changes in retinal microvascular diseases. The study revealed that MVNs made up of endothelial cells (ECs) only were unstable or, in some monocultures of ECs, failed to form. However, when pericytes were added, the vascular area increased, and in the tri-culture condition, basal membranes formed, reflecting the in vivo architecture and function of the retinal vasculature. Diabetic stimulation of the iBRB-on-chip induced vascular regression and pericyte loss, mirroring changes in diabetic retinopathy. Immunofluorescence staining and transcriptomic analysis of these alterations revealed the differential expression of genes involved in vascular instability and pro-inflammatory pathways, underscoring the platform’s potential for studying therapeutic strategies for diabetic retinopathy [55].

In most developed countries, age-related macular degeneration (ADM) is the leading cause of blindness in people over 65 years. This pathological process involves the irregular secretion of vascular endothelial growth factor (VEGF), a key driver of angiogenesis. In a study by Chen et al., shown in Figure 4C, a microfluidic device was designed to study AMD, focusing on choroidal neovascularization. The device consists of two PDMS layers separated by a porous membrane, with ARPE-19 cells seeded in the upper layer, and human umbilical vein endothelial cells (HUVECs) in the lower layer. This setup simulates Bruch’s membrane, which separates the retina from the choroid in vivo. To quantify the possible invasion of HUVECs by ARPE-19 cells, the authors analyzed the growth area of both the HUVECs and the ARPE-19 cells in four different conditions. Figure 4D,E show graphs in which the growth area of the two types of cells cultured on the chip was quantified at different times in a control situation, in the presence of low glucose concentrations in the culture medium, and after the addition of CoCl_2_ to induce cellular hypoxia. In particular, Figure 4D shows HUVEC growth, while Figure 4E displays the growth of ARPE-19 cells in monoculture. A reduction in ARPE-19 growth after 14 h was observed, but this was not as pronounced as for HUVECs. The permeability tests further indicated that the ARPE-19 monolayers were still present even under low-glucose or hypoxic conditions, leading the researchers to hypothesize that the reduced growth of ARPE-19 cells was due to invasion by HUVECs, which caused the detachment of ARPE-19 cells. This provided critical insights into the pathophysiology of AMD [56].

These advancements in BRB-on-chip models provide a robust platform for studying retinal diseases, optimizing drug delivery, and developing novel therapeutic strategies. By replicating the structural and functional properties of the BRB, these systems hold great promise for the development of more effective treatments for retinal disorders.

## 4. Skin-on-Chip

The skin is the body’s largest organ and serves as a critical protective barrier against environmental factors, pathogens, and chemical exposure. It consists of three primary layers, which are, from the outermost to the innermost, the epidermis, the dermis, and the hypodermis. The epidermis comprises keratinocytes, melanocytes, and immune system cells such as Langerhans cells. The dermis contains blood vessels, lymph vessels, sensory nerves, sebaceous glands, sweat glands, hair follicles, and fibroblasts, while the hypodermis consists of adipose tissue, lymphatics, sensory nerves, collagen, and larger blood vessels [55,57].

Organ-on-chip models have been developed to replicate the skin’s complex structure with its vascular, immune, and nervous systems, incorporating cellular heterogeneity, mechanical stimuli, and cell–cell and cell–matrix interactions. Traditional in vitro skin models lack key components such as vascularization, immune responses, and sensory functions [55]. Recent advances in the field of chips with integrated skin have addressed these limitations, allowing for a better simulation of physiological and pathological conditions. The literature presents various skin-on-chip platforms, ranging from simple two-layer models, with a porous membrane integrated to represent the dermis layer of the skin [57], to more complex devices that incorporate multiple layers to simulate the full structure of the skin [57,58]. These advanced models often include matrices made from hydrogels or samples of excised mouse or rat skin. Additionally, some platforms feature a two-layer design with an integrated magnet that generates forces of attraction and repulsion to stretch the cells, effectively mimicking the formation of wrinkles [58,59]. To obtain a skin model closer to the skin in live organisms, an air–liquid interface (ALI) could be included in the platform to expose the outermost cell layer of the epidermis to air, while the dermis layer is exposed to the culture medium or the vascular system.

From the point of view of the various types of cells integrated in SoC devices, cells that recapitulate the epidermis and dermis are very common, but in some works, we also find vascular endothelial cells to imitate vascularization, immune cells [60] that detect external insults, and sensory neurons, which make the cellular microenvironment of the skin-on-chip more complete and realistic [59].

**Figure 5:**
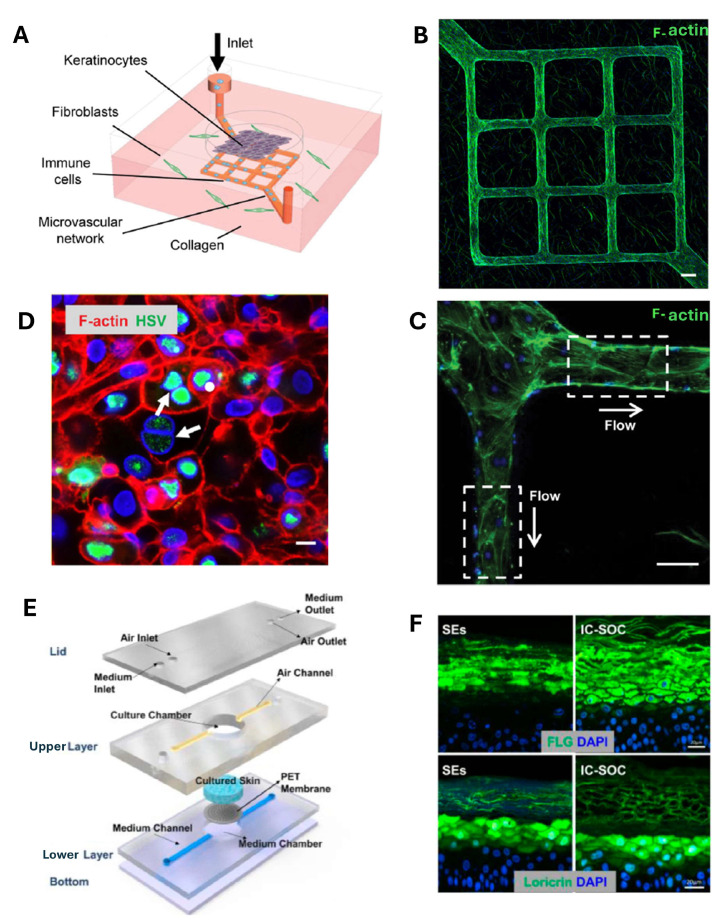
(**A**) Skin-on-chip image by Sun et al. [62] representing the design of the device and the cellular components inside it. (**B**), (**C**) Confocal microscopy images showing (**B**) the presence of the endothelial microvascular network in the grid and the presence of collagen outside the grid, (**C**) an enlargement of part of Figure (**B**), displaying the direction of flow indicated by the white arrows. In both images, the actin filaments are colored in green, and the cell nucleus is in blue (DAPI) (Scale bar 100 µm) [62]. (**D**) Confocal microscopy image showing infection in the epidermis by herpes simplex virus (HSV) in the skin-on-chip. HSV is shown in green, and the actin filaments are in red (scale bar 20 µm) [62]. (**E**) Exploded view of the skin-on-chip system realized by Quan et al. [67]. (**F**) Fluorescence images show the comparison between the expression of filaggrin (FLG) and that of loricrin (stratum corneum proteins) in the static skin equivalent (SE) model and the interface-controlled skin-on-chip (IC-SoC) (scale bar 20 µm) [67].

Skin-on-chip models have been developed to investigate viral infections, such as herpes simplex virus (HSV) infection, and sensory functions involving nerve fiber interactions [61,62]. For example, Sun et al. integrated microfluidics with vascularization to simulate HSV infection and study host immune responses and the effect of antiviral drugs [62]. Their microfluidic device (Figure 5A) contains the main components of the human skin, such as fibroblasts, keratinocytes, collagen, immune system cells, and endothelial cells. To characterize the vascular endothelium within the chip grid and the microenvironment outside the grid after two weeks of dynamic culture, immunofluorescence staining with phalloidin was performed to identify F-actin filaments, as shown in Figure 5B. Furthermore, in Figure 5C, it can be seen that there was an alignment of the cytoskeleton of the endothelial cells with the direction of the flow, suggesting that the cells responded to shear forces. To simulate a tissue microlesion that would facilitate the entry of the virus into susceptible epidermal cells, mechanical destruction was carried out on the epidermis with a dermatological punch, and then the virus was introduced. HSV infection mainly manifested in the keratinocytes, showing pathomorphological characteristics such as enlargement of the nucleus, formation of multinuclei, and marginalization of chromatin, as shown by the white arrows in Figure 5D.

Other researchers created skin-on-chips integrated with nerve fibers, which are present in vivo and are important for the perception of pain, temperature, and mechanical stimuli. In their model, Martorina et al. incorporated neurites extending from the dermis into the epidermis, allowing for sensory function studies using stimulation with capsaicin, which is a transient receptor protein-villanoid-1 (TRPV1) channel agonist. The use of this compound resulted in the formation of calcium currents that showed the activation of the sensory function in the epidermis [63].

Notably, the skin also has its microbiome, which contributes to protecting it from pathogens and to the development of the immune system. It has been seen that in various areas of the skin, different types of bacteria lead to different types of diseases [64,65,66]. Quan et al. [67] designed an interface-controlled skin-on-chip (IC-SoC) to study bacterial infections, particularly *Propionibacterium acnes* (*P. acnes*), which contributes to acne development. Their microfluidic device consists of three PDMS layers: a bottom layer for culture medium flow, a porous PET membrane, and an upper layer for air exposure and cell culture (Figure 5E). The cells used in this platform are immortalized human keratinocytes (HaCaT) in a solution of type I collagen hydrogel and dermal fibroblasts. The presence of airflow promotes keratinocyte stratification and differentiation, mimicking the in vivo conditions. The immunofluorescent staining of some structural proteins such as loricrin and filaggrin in the stratum corneum of the epidermis in the IC-SoC was significantly greater than in static skin equivalents, as shown in Figure 5F, suggesting that under dynamic culture conditions, the skin barrier function is more intact, and this was also confirmed by TEER readings. In the IC-SoC, the stratum corneum is thicker and presents greater structural integrity of the dermal–epidermal junction (DEJ) than the static model. *Propionibacterium acnes* produced inflammation on the surface of the skin barrier formed in the chip and allowed for simulating a pathological condition occurring in vivo. The efficacy of two drugs used in acne treatment, namely, polyphyllin H and dexamethasone, was also tested. These two drugs were found to reduce the effect of pro-inflammatory cytokines, confirming previous studies carried out in vitro. Therefore, drug testing demonstrated the platform’s potential for assessing anti-inflammatory treatments [67].

These advancements in skin-on-chip technology offer valuable platforms for disease modeling, drug testing, and personalized medicine, providing new opportunities for studying skin pathophysiology under controlled microfluidic conditions.

## 5. Cornea-on-Chip

The cornea is the outermost layer of the eye, serving as the primary protective barrier against environmental damage, pathogens, and dehydration, while playing a crucial role in refracting light to facilitate vision. It consists of five distinct layers: the epithelium, Bowman’s layer, the stroma, Descemet’s membrane, and the endothelium. The corneal epithelium is of particular importance, as it forms a tight barrier to regulate fluid and ion transport, preventing microbial invasion and maintaining transparency. The transcorneal route represents the main route for the administration of ophthalmic drugs, which are, predominantly, for topical use. Therefore, having a model that closely simulates the physiology of the cornea in vivo can help in the screening of ophthalmic drugs for local use.

Corneal diseases such as keratitis, corneal ulcers, and dry eye syndrome can significantly impact vision and quality of life. Traditional models for studying corneal pathophysiology include in vitro cell cultures and animal models. Animal models, such as rodent models, present significant differences in their visual apparatus compared to humans; for example, they do not possess the fovea, a retinal structure present in humans, and 85% of their optic nerves decay on the other side of the brain compared to humans. An animal model that best simulates the human eye is the eye of monkeys, which are, however, complicated to breed [68,69]. Today, 2D cell cultures and 3D models are more commonly used, but have limitations and fail to replicate the full complexity of the corneal microenvironment.

**Figure 6:**
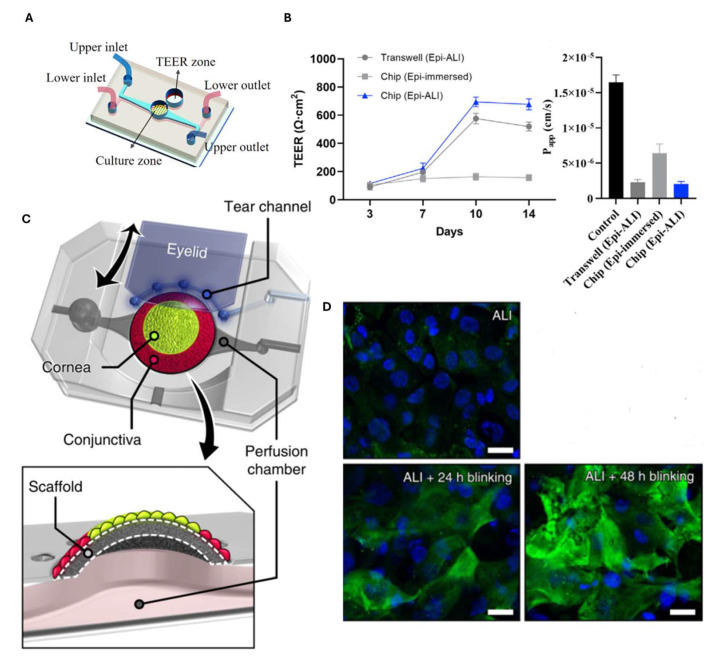
(**A**) Graphic representation of the cornea-on-chip by Yu et al. [75]. (**B**) Graphs of TEER readings (left) and measurements of the permeability coefficient (right) of the corneal epithelium in three different conditions [75]. (**C**) Image of the eye-on-chip by Seo et al. [76]. (**D**) Fluorescence microscope images showing corneal epithelial cells grown under three different conditions: at the air–liquid interface (ALI), with ALI + 24 h of simulated blinking and ALI + 48 h of simulated blinking, to simulate the environment of the organ in vivo (Scale bar 20 µm) [76].

Cornea-on-chip platforms have emerged as promising alternatives by incorporating physiological and biomechanical cues to better simulate the human cornea [13,70,71,72,73,74]. In this respect, Yu et al. developed a cornea-on-chip system to investigate wound healing and epithelial repair mechanisms through extracellular vesicles. Specifically, they designed a microfluidic chip composed of two PDMS layers, each containing a microfluidic channel, with a central circular culture zone to mimic the structure of the human cornea. This zone includes a porous polycarbonate membrane coated with extracellular matrix components (Figure 6A). The device also provides the possibility of measuring transepithelial electrical resistance (TEER) to assess barrier integrity. Human corneal epithelial cells (HCEpi) were cultured in the upper channel, while human corneal endothelial cells (HCEnd) were maintained in the lower channel. Furthermore, to simulate the ocular surface, the HCEpi cells were cultured both in immersion conditions (Chip Epi-immersed) and in air–liquid interface conditions (Chip Epi-ALI), as well as in Transwell Epi-ALI systems for comparison. Figure 6B on the left shows a graph with TEER values as a function of time: the permeability of the HCEpi cells was measured in three different conditions, i.e., Transwell Epi (epithelial)-ALI (air–liquid interface), Epi-immersed chip, and Epi-ALI chip on days 3, 7, 10, and 14. Figure 6B, on the right, on the other hand, displays the permeability coefficient (P_app_) of the corneal epithelium evaluated using 5 kDa FITC-dextran in three different situations (Transwell Epi-ALI, Epi-immersed-ALI chip, and a control situation without the cells). Both graphs show that cell permeability and, therefore, barrier integrity was greater in the Epi-ALI chip, compared to the other two conditions. After confirming the formation of a functional corneal barrier, a controlled corneal wound was induced, and the wound healing process was evaluated upon treatment with vesicles derived from mesenchymal stem cells. These vesicles, known for their anti-inflammatory properties, modulate cytokine expression by promoting the release of anti-inflammatory factors while reducing the levels of pro-inflammatory cytokines, such as matrix metalloproteinase-2 (MMP-2), which was specifically analyzed in this study [75].

Cornea-on-chip platforms have been also used to mimic blinking and study its mechanism as well as to study dry eye syndrome, a condition characterized by tear film instability and inflammation. A contribution on this topic came from Seo et al., who created an in vitro dry-eye-syndrome model that features a 3D cell culture scaffold with primary human keratinocytes to represent the subepithelial stroma within a hydrogel. As shown in Figure 6C, the scaffold is attached to a perfusion chamber and a biomimetic eyelid that can be mechanically operated. The conjunctival and corneal epithelia were reproduced on the scaffold using a 3D cell modelling technique that allows for the assembly of different multilayer cell structures. The authors also investigated how engineered blinking has an effect on the differentiation of corneal epithelial cells in the presence of an air–liquid interface (ALI). In Figure 6D, the upper left image shows a situation where cells were subjected to submerged culture for two days, followed by three days of ALI culture without simulated blinking. Only the nucleus can be seen in blue (DAPI). In the lower image on the left, the cells were in submerged culture for two days, followed by one day of ALI culture without simulated blinking and one day of ALI+ blinking; cytokeratin 3/12 (CK-3/12) which is a protein specific to differentiated corneal epithelial cells is highlighted in green. This device may pave the way for the study of human ocular mechanisms and the discovery of new ophthalmic drugs [76].

Another reported application of the cornea-on-chip concerned the investigation of bacterial keratitis. This is an inflammation of the cornea caused by bacterial invasion, often following trauma, and in extreme cases, can even lead to the loss of sight. This pathology requires prompt medical treatment, but the improper use of antibiotics has contributed to an increase in drug resistance; therefore, there is an increased interest in identifying new therapeutic strategies for bacterial keratitis. Cornea-on-chip platforms have proven to be an interesting and innovative tool for this purpose. In the work of Deng et al., immortalized human corneal epithelial cells and primary human corneal fibroblasts were co-cultured on a porous polydimethylsiloxane membrane to develop a cornea-on-chip model, reconstructing an epithelial tissue with a realistic and reproducible structure. By inducing controlled epithelial damage and bacterial infection, an in vitro model of bacterial keratitis using *Staphylococcus aureus* was created. The efficacy of antibiotics such as levofloxacin, tobramycin, and chloramphenicol was evaluated by simultaneously monitoring the responses of bacteria and corneal cells, highlighting the differences between the drugs in terms of bactericidal activity, reduction in cell apoptosis, and prevention of scar formation. This corneal-on-a-chip model provides a cutting-edge system for testing ocular antibiotics, allowing for a comprehensive evaluation of their effects on corneal cells and the overall corneal structure [77].

All these achievements demonstrate that cornea-on-chip technology provides a robust platform for studying corneal diseases, testing ophthalmic drugs, and exploring novel treatment strategies. By integrating key physiological parameters such as tear flow, mechanical stress, and multi-cellular interactions, these models offer significant improvements over traditional approaches, paving the way for precision medicine in ophthalmology.

## 6. Airway-on-Chip

Another biological barrier is located in the respiratory system, which includes the nose, the pharynx, the larynx, the trachea, the lungs, the pleura, the bronchi, and the bronchioles. This barrier not only allows for gaseous exchanges between the external environment (oxygen) and the human being (carbon dioxide) but also protects from airborne particles, pathogens, and environmental pollutants that can settle in the airways. The airway epithelium, composed of ciliated epithelial cells, goblet cells, and basal cells, serves as the first line of defense against these external agents. This barrier is crucial for mucociliary clearance, immune responses, and gas exchange. Ciliated epithelial cells together with a layer of mucus trap pathogens and particles, preventing their access to the lungs, while macrophages on the surface of the alveoli phagocytose potentially dangerous microorganisms. Disruptions in the airway barrier can lead to respiratory diseases such as chronic obstructive pulmonary disease (COPD), asthma, and cystic fibrosis [78,79,80].

Traditional in vitro models, including 2D cultures of airway epithelial cells, fail to accurately recapitulate the complex structure and function of the respiratory epithelium, in particular to mimic the mechanical stimulus of respiration and the air–liquid interface (ALI). Furthermore, 2D models are unable to simulate the interstitial flows produced by the vascular system, which are important for cell–cell communication and for the formation of concentration gradients [81]. Similarly, animal models exhibit physiological differences that limit their translatability to human conditions, for instance, the inability, when exposed to smoke, to reproduce disabling lung disease. To address these challenges, airway-on-chip models have been developed to provide a more physiologically relevant platform for studying lung diseases, drug responses, and environmental toxicology, integrating mechanical stimuli and the presence of an air–liquid interface (ALI) or the alveolar–capillary interface (ACI). Moreover, they can also be integrated with biosensors that can detect analytes (biomolecules and microorganisms) and thus monitor the progress of pathologies [27,28,29,30].

One of the earliest airway-on-chip models was developed by Huh et al., incorporating human alveolar epithelial cells, lining a porous fibrin–collagen membrane, and human microvascular endothelial cells, cultured at the air–liquid interface (ALI), within a microfluidic device consisting of two vertically stacked polydimethylsiloxane layers. The air–liquid interface is used to mimic the lining of the alveolar airspace, while compartmentalization is used to reproduce the fluid flow and allow for the administration of substances into the endothelial and epithelial channels, separately. Furthermore, the device is characterized by lateral chambers, which are connected to a vacuum pump that applies a cyclical alteration to deform the membrane and, therefore, also the epithelial cells above it, recreating the mechanical distortion associated with the respiratory movements. This model successfully mimics key physiological features of the lung, including mucus production, ciliary beating, and barrier function. The novelty of this work consists in using the platform to investigate the toxicological effects on the lungs caused by 12 nm silica nanoparticles simulating the ultrafine particles present in the air reaching the alveolar epithelial cells. The authors found that the nanoparticles stimulated lung inflammation through the production of pro-inflammatory cytokines, such as TNF-α, and the upregulation of adhesion molecules such as ICAM-1, which recruit leukocytes, and this was also accentuated by the respiratory movements reproduced in the device. This platform was further used to study the inflammatory responses to pathogens and the effects of mechanical forces on lung epithelial integrity [82].

Benam et al. modified this concept by developing a human small airway-on-chip model that integrated alveolar epithelial cells and lung fibroblasts. Their model allowed for the investigation of COPD by exposing the cells to cigarette smoke, providing insights into inflammation, oxidative stress, and tissue remodeling. The chip demonstrated the ability to reproduce disease progression, which makes it a valuable tool for drug testing and biomarker discovery [83].

**Figure 7:**
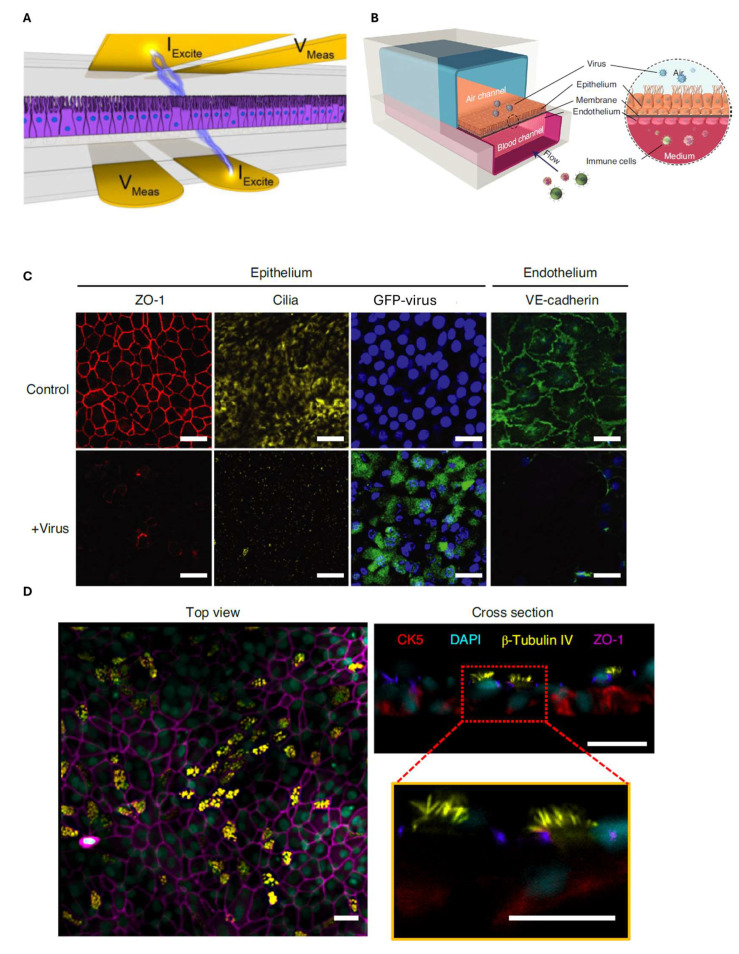
(**A**) Schematic representation of the TEER-chip [84]. (**B**) Drawing of a cross section of the airway chip containing the air–liquid interface (ALI) [87]. (**C**) Immunofluorescence images showing cell–cell tight junctions containing ZO-1 (red), cilia (yellow) in the epithelium, and VE-cadherin in the endothelium (green) of the airway chip both in the absence (top) and in the presence (bottom) of influenza virus, which expresses green fluorescent protein (GFP) [87]. (**D**) Immunofluorescence images showing a cross section of the pseudostratified epithelial layer of the human airway with cells expressing cytokeratin 5 (CK-5) in red and beta-tubulin IV in yellow, as well as DAPI in blue and ZO-1 in purple [87] Scale bars 50 um.

In another work, Henry et al. introduced electrodes in airway-on-chip devices having two channels separated by a porous PET membrane. A current was applied between two electrodes (I_excite_) positioned above and below the cell culture, and the potential drop between two other electrodes (V_mas_) was measured, as shown in Figure 7A. In this way, it was possible to carry out real-time measurements of the transepithelial electrical resistance of a monolayer of primary human airway epithelial cells (hAEC) for a maximum of 62 days of culture [84]. This design provides a valuable approach for evaluating airway barrier integrity, also in terms of inflammatory responses and in the case of exposure to airborne pollutants, including particulate matter and industrial chemicals.

Khalid et al. developed a platform for lung cancer on a chip made of two layers of glass covered with indium tin oxide (ITO) electrodes for TEER measurements to assess the cytotoxicity of certain drugs used to treat lung cancer. In addition, their chip was also connected to a sensor for pH monitoring. In the upper microfluidic channel, human lung adenocarcinoma cells (NCI-H1437) were seeded and treated with doxorubicin and docetaxel, which are two anti-cancer drugs. Studies have shown that doxorubicin exhibits higher cytotoxicity than docetaxel, as demonstrated through TEER impedance measurements, cellular index (CI) assessment, pH monitoring, and cell viability assays. This sensor-integrated microfluidic device offers a versatile platform for evaluating the cytotoxic effects of novel drugs and compounds in real time [85].

Another application of airway-on-chip technology involves modeling viral infections such as influenza and SARS-CoV-2 infections [86,87,88]. Si et al. [87] developed a lung-on-chip system that replicated the infection dynamics of respiratory viruses (including SARS-CoV-2). Their model enabled the real-time monitoring of viral replication, immune responses, and cytokine secretion. The ability to study host–pathogen interactions in a controlled environment has significant implications for antiviral drug screening and vaccine development [87,88]. Endothelial cells and primary basal stem cells of the human lung bronchial airway epithelium were, respectively, seeded in the upper (air) channel and in the lower (blood) channel (Figure 7B). To test whether the device could be used to identify new treatment strategies against pandemic respiratory viruses, it was initially tested with influenza A virus. Using immunofluorescence techniques, in the absence of the virus, the authors observed the formation of tight junctions containing ZO-1 (in red in Figure 7C and in purple in Figure 7D) and of cilia made of β-tubulin (in yellow) (Figure 7C,D), with epithelial barrier properties such as permeability and mucus production similar to those observed in human airways in vivo. Furthermore, the underlying human pulmonary microvascular endothelium also formed a continuous planar monolayer with cells connected by adherens junctions containing VE-cadherin (green) (Figure 7C). In contrast, in the presence of a virus labeled with GFP, there was damage to the epithelium and the endothelium, as suggested by the loss of cilia and adherens junctions. After creating the air–liquid interface (ALI), infection by different influenza strains such as H5N1, H3N2, and H1N1 was evaluated by fluorescence microscopy, reporting epithelial damage, destruction of occluding junctions, and loss of cilia. H3N2 and H5N1 were the viruses causing a more severe pathogenesis, with a greater release of cytokines and chemokines. Then, the authors compared the immune response occurring in the chip and tested drugs to block the entry of the coronavirus.

These advancements in airway-on-chip technology offer a versatile and physiologically relevant approach to studying lung diseases, pathogen–host interactions [89], and environmental toxicology. By integrating biomechanical forces, immune components, and biosensors, these microfluidic platforms provide an innovative tool for disease modeling, drug testing, and personalized medicine applications [87,88].

## 7. Gastrointestinal Barrier-on-Chip

The gastrointestinal (GI) barrier plays a fundamental role in maintaining intestinal homeostasis by regulating the exchange of nutrients, water, and electrolytes, while preventing the entry of pathogens, toxins, and allergens [90]. This barrier is composed of a monolayer of intestinal epithelial cells interconnected by tight junctions, along with mucus-producing goblet cells and immune components that contribute to the host defense. A further protective action is also exerted by the intestinal microbiota [91] and the gut-associated lymphoid tissue (GALT), which represents the immune system in the digestive tract and has the complex task of tolerating the presence of commensal bacteria and responding to pathogenic microorganisms [33,92,93,94]. Disruptions in the GI barrier have been linked to various diseases, including inflammatory bowel disease (IBD), celiac disease, and colorectal cancer.

Traditional in vitro models, such as Transwell systems, lack the complexity needed to replicate the dynamic interactions within the intestine. In addition, 2D cultures cannot mimic intestinal peristalsis and do not replicate the 3D architecture of the organ. Similarly, animal models exhibit interspecies differences that limit their applicability to human GI physiology. To overcome these challenges, gastrointestinal barrier-on-chip models have been developed to mimic the structure and function of the intestinal epithelium under physiologically relevant conditions [95,96,97,98]. Most of the gut-on-chip models described in the literature can be divided into two types: platforms with two layers that simulate the intestinal endothelial and epithelial compartments, separated by a membrane coated with extracellular matrix proteins, such as the one created by Kim et al., Jalili-Firoozinezhad et al., Shah et al., and Jeong et al., and platforms consisting of three channels on a single plane, such as the one created by Beaurivage et al., which has a central channel used to set up the extracellular matrix and two channels used to simulate the endothelium and the intestinal epithelium.

Kim et al. designed one of the first gut-on-chip platforms, incorporating human intestinal epithelial cells exposed to fluid flow and cyclic mechanical strain to simulate peristalsis. They studied the interaction between microbes and human intestinal epithelial cells using *Lactobacillus rhamnosus* GG (LGG) and measured beta-galactosidase activity to study the viability of the microbe, which remained high even in co-culture in the gut-on-chip. Therefore, this model successfully recreates the formation of intestinal villi, the integrity of the barrier, and host–microbiome interactions, which makes it a valuable tool for the study of inflammatory and infectious diseases [99]. Maurer et al. expanded on this concept by integrating a gut-on-chip system with a co-culture of both *Lactobacillus rhamnosus* and *Candida albicans* to study microbiota–pathogen interactions. Their findings demonstrated the role of probiotic bacteria in inhibiting fungal overgrowth and maintaining intestinal homeostasis. This approach provides a promising avenue for investigating microbiome-based therapies for gut disorders [100].

Another important study carried out by Nikolaev et al. focused on the creation of functional, self-organizing mini-intestines. The novelty of this work consists in using a combination of intestinal stem cells to form an epithelium with a spatial arrangement resembling that of intestinal villi and crypts and with an accessible lumen, within a microchannel generated by laser ablation, and surrounded by an extracellular matrix composed of Matrigel and type I collagen, which can be colonized by other cells supporting the intestinal epithelium, such as macrophages or endothelial cells. In addition, these mini-intestines can be perfused through an external pump, which allows for the continuous removal of dead cells. The authors subjected these miniature intestines to epithelial damage to study their regenerative power and also investigated the possibility of using these intestinal models to study long-term parasitic infections [101].

**Figure 8:**
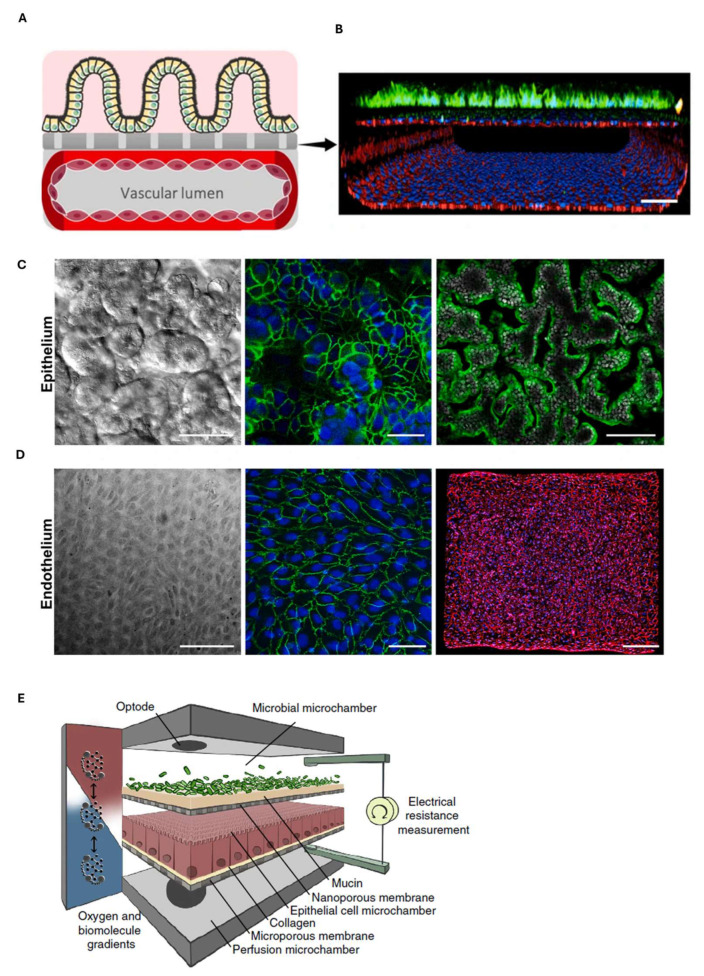
(**A**) Graphic representation of the intestinal villi grown on the membrane of the upper channel and the endothelial lumen formed in the lower channel [102]. (**B**) Confocal microscope image of a cross section of the artificial intestine inside the platform (bar 100 µm) [102]. (**C**) DIC image on the left, showing the morphology of the villi of the intestinal epithelium consisting of Caco-2 cells kept in culture for 5 days in the gut-on-chip (bar 50 µm). Centre and right fluorescent microscopy images showing ZO-1 occluding junctions (green, center, bar 50 µm), villin (green, right, bar 100 µm) and the nucleus in blue (DAPI) [102]. (**D**) Phase-contrast microscopy image of the endothelium kept in culture for 5 days in the gut-on-chip (bar 50 um). Middle and left fluorescence microscopy images show cell junction-associated proteins including PECAM-1 (green, middle, bar 50 micrometers) and VE-cadherin (red, right, 200 um), with nuclei in blue [102]. (**E**) Drawing of the device by Shah et al., containing a co-culture of human epithelial cells and gastrointestinal tract bacteria [104].

A two-layer human gut-on-chip was also reported by Jalili-Firoozinezhad et al. (Figure 8A) with a porous membrane separating human epithelial cells in the upper layer from vascular endothelial cells in the lower layer. In Figure 8B, the confocal image shows epithelial villi (green), adherens junctions consisting of VE-cadherin in the endothelium (red), and nuclei (blue), within the device. The cells were kept in culture for 5 days, and then the intestinal epithelium and endothelium were characterized by immunofluorescence techniques. Figure 8C shows epithelial villi characterized by ZO-1 in green, in the center, and by villin in green, in the image on the left, while Figure 8D shows the endothelium characterized by PECAM-1 (green, in the center) and VE-cadherin (red, on the left). The authors then induced intestinal damage with gamma radiation to explore the mechanisms underlying radiation-induced gastrointestinal syndrome [102].

The presence of an oxygen gradient in the intestine is another relevant aspect, with values increasing from the inside (the lumen) to the outside (the intestinal mucosa). Its maintenance is important to allow the intestinal barrier to perform its protective functions. Indeed, in the event of a loss of this balance, which can occur through inflammation of the intestine or bacterial infection, free radicals are formed, which are harmful to cells [103]. In this respect, Shah et al. reported a platform (Figure 8E) able to account for this gradient and ensure the crosstalk between intestinal epithelium (Caco-2) and the different types of bacteria living in the gut, which can live under aerobic conditions, such as *Lactobacillus rhamnosus*, or anaerobic conditions, such as Bacteroides Caccae. Oxygen sensors were also integrated here to monitor the oxygen gradient in real time, generated with oxic and anoxic culture media, to allow for the growth of aerobic and anaerobic bacteria. In this way, this platform proved to be a useful tool for better investigating the relationship between gut and microbiota, which is also important for the study of intestinal diseases [104].

Other relevant aspects to investigate concern intestinal diseases, drug absorption through GI barrier-on-chip models, and metabolism through multi-organ-on-chip platforms. Beaurivage et al. developed an intestine-on-chip suitable for investigating mechanisms relevant for inflammatory bowel disease (IBD) [105], including the exposure to immune-relevant inflammatory triggers and anti-inflammatory compounds as well as the elimination of key inflammatory regulators, and for evaluating their influence on the barrier function and the release of cytokines. An unprecedented aspect of this study is the use of direct adenoviral transduction on a chip to conduct knockdown studies of inflammatory molecules such as CCL20, a chemotactic factor for lymphocytes.

To mimic the human stomach, Jeong et al. developed a two-layer membrane-based OoC, seeding epithelial cells derived from human antral organoids (hAOs) in the upper channel and primary gastric mesenchymal stromal cells (gMSCs) in the lower channel. The organoid-derived cells provided gastric stem cells that can differentiate and are an important resource for the formation of the gastric mucosal barrier. In addition, the presence of fluid flow and communication between the two cell lines mimics gastric homeostasis and mucosal function. After successfully reproducing the gastric microenvironment, the authors simulated Helicobacter pylori infection by introducing the bacterium into the upper compartment, while seeding human peripheral blood mononuclear cells (PBMCs) in the lower channel. Due to the crosstalk between the gastric cells and the immune system cells, the results showed an increased expression of cytokines and nuclear factor KB (NF-KB) compared to those obtained in in vitro assays, demonstrating that this platform can be used to study gastric defense mechanisms and develop pharmacological therapies [106].

These advancements in gastrointestinal barrier-on-chip technology offer a physiologically relevant platform for studying gut health, disease mechanisms, and therapeutic interventions. By incorporating biomechanical cues, immune components, and microbial interactions, these systems provide a powerful tool for advancing research in gastroenterology and personalized medicine. 

## 8. Testis-on-Chip

The blood–testis barrier (BTB) is primarily formed by Sertoli cells and occluding junctions and plays a crucial role in maintaining the immune-privileged environment of the testes, regulating the transport of molecules, and ensuring proper spermatogenesis. This barrier separates the blood vessels from the seminiferous tubules of the testicles to prevent the reflux of immature cells between the basal zone, where the primary spermatocytes and spermatogonia are found, and the adluminal zone, where the secondary spermatocytes are located. The BTB protects developing germ cells from harmful substances and immune attacks. Its dysfunction has been linked to male infertility, testicular cancer, and endocrine-disrupting chemical exposure [107,108,109].

Conventional models based on 2D cell cultures and animal models fail again to replicate the complexity of the testicular microenvironment or present interspecies differences that limit their translatability. To address these challenges, testis-on-chip models have been developed to better mimic the physiological and functional characteristics of the testis in vitro [110,111,112]. Furthermore, they allow for introducing physical and chemical stimuli, continuously supplying nutrients and hormonal stimuli, and removing metabolic products through perfusion of the chip in a simulated vascular system [113]. They could be useful to better study the process of spermatogenesis and provide a solution for pre-pubertal cancer survivors for whom there is no hope of becoming parents.

**Figure 9:**
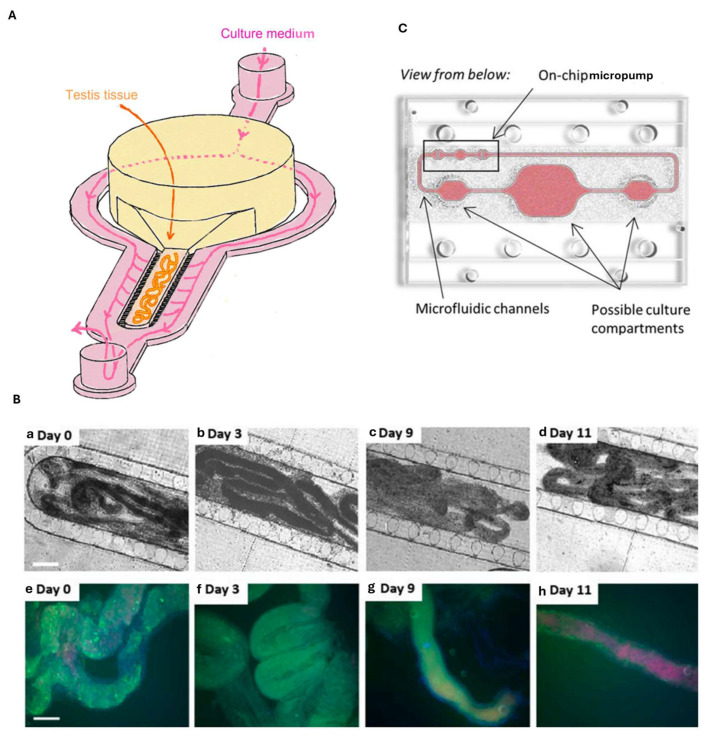
(**A**) Sketch representation of the testis-on-chip device developed by Sharma et al. [107]. (**B**) Images (**a**) to (**d**) are from light microscopy and show the maintenance of tissue integrity in the chip up to day 11. Images (**e**) to (**h**) show a live/dead assay, with calcein (green) highlighting live cells, and propidium iodide (red) indicating dead cells. This assay was used to highlight cell viability in the primate seminiferous tubules on the chip [107]. (**C**) Bottom view of the multi-organ platform including liver and testes by Baert et al. [114].

Sharma et al. [107] developed a microfluidic testis-on-chip system incorporating an ex vivo tissue culture of seminiferous tubules from prepubertal marmosets. The chip has a central chamber containing the tissue (yellow) surrounded by a perfusion channel representing the vascular system (pink), and the two structures are separated by a series of pillars, as shown in Figure 9A. Their platform successfully recreated the structural and hormonal environment required for spermatogenesis, demonstrating that the seminiferous tubules maintain functionality under controlled conditions. In this study, the authors demonstrated the platform’s ability to support ex vivo tissue cultures of primate seminiferous tubules, monitoring the culture subjected to continuous perfusion for 11 days. As shown in the Figure 9–Coptical microscopy images in Figure 9B the integrity of the tissue was not damaged. A vitality/death test was also carried out at different time points (3–9–11 days) on the culture of human seminiferous tubules outside the chip, with calcein indicating live cells (green), and propidium iodide (red) indicating dead cells. They observed that the seminiferous tubules remained viable for 9 days inside the chip, and then dead cells were observed in some samples, as shown in Figure 9B(e–h). Next, marmoset tissues were stimulated with high and low doses of gonadotropins, hormones produced by the adenohypophysis that regulate the development of the male and female genital organs. Then, the tissue response was analyzed through histological analysis and by determining the serum testosterone and estradiol levels with ELISA tests. When stimulated with high doses of gonadotropins, the epithelium of the seminiferous tubules appeared more organized and more complete, with Leydig cells, Sertoli cells, and germ cells. The authors demonstrated the importance of hormonal stimulation for the endocrine capacity of the seminiferous tubules of prepubertal marmosets and the ability of the OoC platform to integrate the vascular system to better mimic the physiology of the testicular apparatus [107].

Testis-on-chip platforms can also facilitate systematic studies about the effect of drugs and their metabolites at the testicular level. Metabolic activation by enzymes such as cytochrome P450 (CYP450) occurs for many drugs. Thus, it can be useful to implement a multi-organ platform, in which both the testicular and the hepatic compartments are mimicked. In this respect, Baert et al. [114] designed a human testis-on-chip model which allowed for co-culturing (i) human liver spheroids consisting of HepaRG cells and primary human liver stellate cells and (ii) human testicular organoids obtained from patients with complete spermatogenesis, undergoing bilateral orchiectomy. The chip features a circuit consisting of a larger central compartment for the testis connected via microfluidic channels to two smaller lateral compartments, one for the liver, and the other for the culture medium (Figure 9C), and also features a peristaltic micropump for generating a continuous pulsatile flow. The researchers studied the effect of cyclophosphamide, a chemotherapy drug, on spermatogonia, which are the precursors of spermatozoa. Cyclophosphamide is metabolized by the liver to 4-hydroxycyclophosphamide, enters the cells, and binds to DNA, causing replication inhibition and, thus, cell apoptosis. However, this molecule causes the death not only of cancer cells but also of healthy cells, including spermatozoa, and causes gonadotoxicity. In this experiment, a reduction in germ cells was seen under treatment with cyclophosphamide in the situation where there was a co-culture of germ cells and liver cells, which activate cyclophosphamide. In contrast, in the single-germ-cell culture, more cells survived following the cyclophosphamide treatment. This study shows the importance of multi-organ platforms, especially when testing prodrugs that are metabolized in the liver before acting on the target organs. Without a multi-organ (liver and target organ) design, one could draw physiologically incorrect conclusions, neglecting possible side effects (or benefits) [114].

To study the effects of anti-tumor drugs on testicular tissue, Shen et al. created a microfluidic device consisting of a layer of PDMS containing a chamber, used as testicular tissue, with pillars to allow for better penetration of nutrients and reduce the damage caused by the shear forces of the flow. Fragments of testicular tissue of human origin were inserted between the glass layer and the PDMS layer, and the chip was closed with upper and lower fasteners. Then, after testing its vitality and functionality, the tissue was subjected to the effects of busulfan, a chemotherapy drug known to have toxic effects on germ cells. Immunofluorescence staining techniques were used to observe that the germ cells spermatogonia and differentiated spermatogonial stem cells showed a significant reduction in number, unlike Sertoli and Leydig cells, which were not reduced significantly. These results suggest the device’s potential for reproductive toxicological research and for screening non-toxic compounds [110].

In conclusion, these studies provide insights into the potential of organ-on-chip technology for reproductive toxicology and fertility research. Advancements in testis-on-chip technology provide powerful tools for studying male reproductive health, fertility preservation, and endocrine disruptor effects. By incorporating key physiological parameters such as hormone signaling, cell–cell interactions, and real-time monitoring capabilities, these models will pave the way for improved reproductive research and personalized medicine applications, taking into account also metabolic effects.

## 9. Placenta-on-Chip

**Figure 10:**
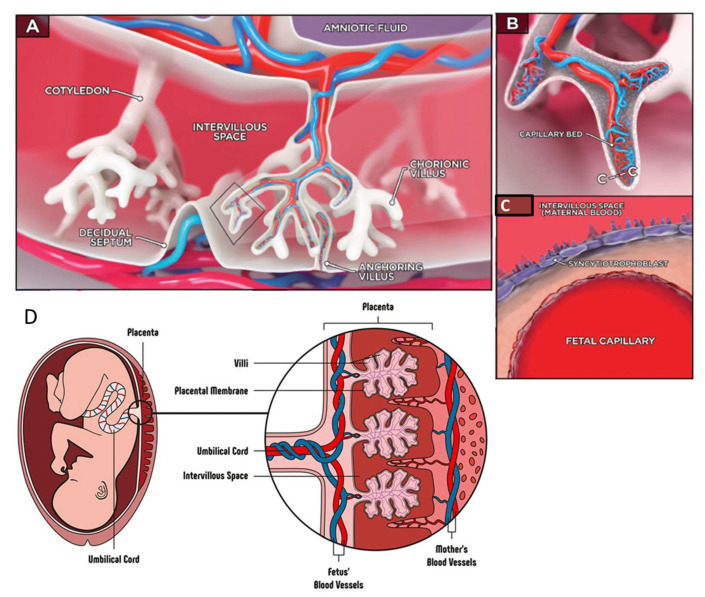
(**A**) Three-dimensional image of some elements that make up the human placenta. Cross section of the cotyledon, chorionic villus, and anchor villus [119]. (**B**) Zoom of a cross section showing the fetal capillaries contained in the chorionic villus [119]. Section (**C**) The placental barrier separates the fetal capillaries from the maternal intervillous space and consists of the endothelial cells of the fetal capillaries, a layer of interstitial tissue, and the syncytiotrophoblast [119]. (**D**) On the right, image of a fetus inside the placenta, connected to it via the umbilical cord. On the left, schematic representation of a part of the placenta, showing maternal blood vessels, chorionic villi, intervillous space, fetal blood vessels, and umbilical cord [124].

The placenta is an organ that forms in the uterus during pregnancy and has several functions. It facilitates nutrient and gas exchange between the mother and the fetus and serves as a selective barrier to protect the developing fetus from harmful substances, while allowing for the passage of antibodies. We can define the placenta as a kind of barrier consisting of several layers, which are composed of different types of cells. The placenta has a complex structure composed of cells that originate from the blastocyst and are subtypes of trophoblasts, such as syncytiotrophoblasts, extravillous trophoblasts, trophoblast giant cells, and villous cytotrophoblasts (Figure 10A–D). In addition, there are also decidua cells originating from the uterine endometrium and placental macrophages [34,115,116,117,118]. Trophoblast cells, endothelial cells, and a basement membrane, form the maternal–fetal interface. The proper function of the placental barrier is essential for fetal development, and its dysfunction has been associated with pregnancy complications such as preeclampsia, intrauterine growth restriction, and gestational diabetes.

Exposure of the fetus to drugs or other molecules is a major problem during pregnancy. Traditional models for studying placental transport, drug effects, and pathologies are limited by ethical concerns, interspecies differences, and the inability to fully replicate the dynamic environment of the maternal–fetal interface. Animal tests can provide inconclusive results due to the different structure of the human placenta compared to the animal placenta [119]. Placental transport studies have also been performed in humans, but are time-consuming and always represent a risk to the fetus. Cell cultures are not effective enough for placental studies, as they cannot faithfully reproduce the dynamic and mechanical microenvironment necessary for placental function and physiological tissue architecture [116]. Studies on cell–cell and cell–matrix interaction have also been carried out with Transwell systems but failed to provide information on the biomechanical control of cellular properties [112]. To address these challenges, placenta-on-chip platforms have been developed as physiologically relevant in vitro models to study placental function and drug transfer across the placental barrier [118,119,120,121,122,123].

**Figure 11:**
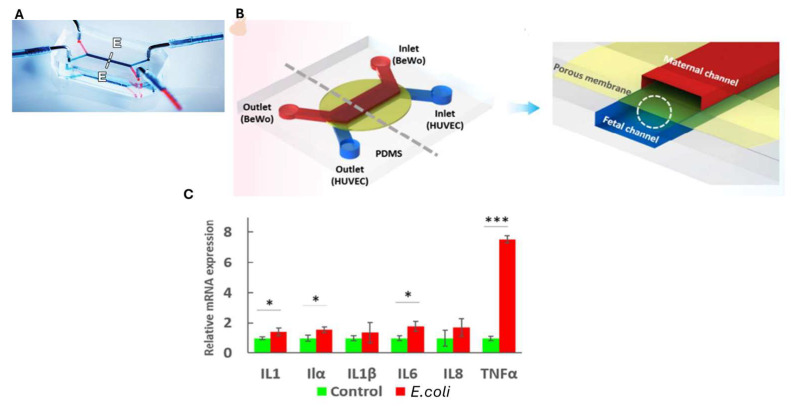
(**A**) Photo of the micro-engineered model representing the placenta-on-chip [119]. (**B**) Picture of the device realized by Zhu et al. on the left, and enlarged cross section of the chip on the right [127]. (**C**) Graph illustrating a quantitative real-time PCR showing the relative expression of inflammatory cytokines with and without *E. coli*. * *p* < 0.05, *** *p* < 0.001 [127].

Among the various models of placenta-on-chip described in the literature, there is a planar model that has channels for the cells and a channel for the extracellular matrix, connected by pillars or microchannels; in addition, there is a two-layer model with the channels separated by a porous membrane, to imitate the maternal–fetal interface. As for the type of cells most commonly used, these include endothelial cells from the umbilical vein (HUVECs), placental vascular endothelial cells (HPVECs), and BeWo cells from embryoid bodies cultured with a trophoblast cell line [121,123]. Blundell et al. [119] developed a placenta-on-chip model (Figure 11A) incorporating human trophoblast and endothelial cells cultured on the opposite sides of a semi-permeable polycarbonate membrane coated with fibronectin. Specifically, human trophoblasts were seeded in the upper layer (maternal), and human placental endothelial villous cells in the lower layer (fetal), under continuous perfusion to ensure the formation of a trophoblast–endothelium interface similar to that present in the human placenta in vivo. This model mimics the placental barrier function and allowed for the real-time monitoring of the transport of a drug used in pregnancy to treat diabetes, i.e., glyburide. After verifying the presence of a functional and structural barrier by immunofluorescence staining, permeability tests, and TEER, Blundell et al. investigated glyburide transport both in the presence and in the absence of trophoblasts on the membrane. The amount of drug perfused remained unchanged in the maternal compartment (upper layer) without cells. In contrast, in the presence of trophoblast cells, the glyburide concentration was lower, suggesting the uptake of the drug by the cells in the upper layer. This platform allows for gaining insights into the selective permeability of the placental barrier and investigating the role of transporters such as breast cancer resistance protein (BCRP) in drug efflux. However, this model represents the structure of the human placenta at the final stage of pregnancy and is not sufficient to investigate drug transport during the first months of gestation, because the placental tissue has a different architecture during this stage than during the last months.

One of the complications that can occur during pregnancy is preeclampsia, characterized by high blood pressure and damage to organs such as the liver and the kidneys [125]. One of the main problems in the study of preeclampsia is the difficulty of modeling human placental physiology in classic laboratory systems. To overcome this limitation, microfluidic placenta-on-chip platforms can be used. Ghorbanpour et al. created a placenta-on-chip to investigate the expression of the binding protein FK506 (FKBPL) and galectin 3 (Gal-3) that are associated with vascular dysfunction in preeclampsia and to study how the formation of the placental vascular network can vary during this pathology. An inflammatory situation was created by stimulating the endothelial cells and the trophoblasts with TNF-α, an inflammatory cytokine expressed by these cells during preeclampsia, and the authors saw that the concentration of FKBPL and Gal-3 increased in the microfluidic device compared to that measured in a non-inflammatory condition and that the trophoblasts were responsible for the observed reduced formation of the placental vascular network [126].

Placenta-on-chip can also help to study inflammation of the placenta, which can be caused by bacterial infections and which often leads to preterm delivery of the newborn, the predominant cause of neonatal morbidity and mortality. Placental inflammation is characterized by loss of the placental function and the presence of inflammatory substances that can damage fetal organs. Zhu et al. [127] created a dynamic placenta-on-chip platform to examine the inflammatory reaction to placental bacterial infection triggered by *Escherichia Coli* (*E. Coli*). Their platform consists of an upper channel seeded with human trophoblast cells that mimics the maternal environment, separated through a porous membrane from the lower compartment, seeded with HUVECs (human endothelial cells) and simulating the fetal microenvironment, as shown in Figure 11B. *E. Coli* was inserted into the maternal compartment to simulate infection, and human macrophages (THP-1) on the trophoblast layer of the placenta-on-a-chip. Figure 11C shows data from a real-time quantitative PCR analysis of inflammatory gene expression in control and *E. coli*-stimulated trophoblasts. The trophoblasts produced inflammatory cytokines including IL-1α, IL-1β, and IL-8, and the maternal macrophages were activated, thus confirming the crosstalk, which occurs in vivo during gestation, between trophoblasts and maternal macrophages. In addition, in case of placental inflammation, there is also an elevated production of inflammatory cytokines in fetal blood vessels. This device can be used to simulate bacterial infections that can occur in the human placenta and as a tool for the discovery of new drugs for the treatment of placental infections. A further upgrade consists in the use of pluripotent stem cells to obtain trophoblasts, as tested by Lermant et al. [22]. Other immune cells such as granulocytes and T cells could be included in the device to make the model more predictive [127].

These advancements in placenta-on-chip technology provide a robust platform for studying placental function, disease mechanisms, and drug safety during pregnancy. By incorporating physiological shear stress, co-culture systems, and real-time monitoring, these models hold great promise for improving maternal–fetal medicine and developing safer pharmacological treatments for pregnant individuals.

**Table 2:** Summary of the main characteristics, cell lines, preclinical applications, and various problems of the organs-on-chips discussed above.

Barriers-on-Chips	Key Features	Critical Issues	Fabrication Method and Channel Size	Cell Lines	Preclinical Applications
Blood–brain barrier-on-chip	Blood–brain barrier generation with endothelial cells (hMEC/D3), astrocytes, and bovine pericytes in a 3D extracellular matrix [18,23,32] Presence of mechanical stimuli to simulate flow in blood vessels [23,32]. Presence of biosensors to monitor the integrity of the blood–brain barrier [18,25,26]	Lack of standardization for quantitative assessment of blood–brain barrier function [26]	Photolithography [49,50] Soft lithography [48,50] Laser cutting [49] Wet etching for interdigitated electrodes [49] 3D printing [48] CHANNEL SIZE 2 mm width, 180 µm height, 20 mm length [49] Lower channel: 70 µm height. Higher channel: 200 µm height, 400 µm width [50] Hollow microchannels: 2 cm length, 1 mm width. Top channels: 1 mm height, 1 mm width. Bottom channel: 2 mm height [48]	Endothelial cells: hCMEC/D3 Astrocytes Bovine pericytes [18,23,32]	Optimization of drug passage through the BBB for studies of neurodegenerative diseases and tumors [18,25,26]
Blood–retinal barrier-on-chip	Generate an endothelial and an epithelial compartment to simulate iBRB and oBRB, respectively [19,83]. Presence of electrodes to monitor the formation of occluding junctions through TEER [54,56]	Difficulty maintaining a cell culture for a long time to study chronic diseases, such as maculopathies [19,83] Lack of standardization makes it difficult to compare different models [19,83]	Photolithography [54,56] Soft lithography [54,56] E-beam evaporation Lift-off process Deep reactive-ion etching (DRIE) [54] CHANNEL SIZE 2 µm width, 4 µm depth [54]	Primary human retinal endothelial cells (HRECs) [54] Human neuroblastoma cell line (SHSY5Y) [54]. Human retinal pigment epithelium cell line (ARPE-19) [54,56] Human umbilical vein endothelial cells (HUVECs) [56] Human retinal astrocytes (HRAs) [55] Human retinal microvascular pericytes (HRP) [55] Human retinal microvascular endothelial cells (HRMVECs) [55]	Study of diabetic retinopathy, age-related retinopathy [54,55] Study of age-related macular degeneration [54] Study of angiogenesis under physiological and non-physiological conditions [56]
Skin-on-chip	Production of the different layers that make up the skin, using heterogeneous cell types [62,67] Presence of cell–matrix interaction [34,67,128] Presence of airflow for keratinocyte differentiation [67]	Lack of important components, such as vascular or immune cells [67] Optimization of culture time and medium composition [63,66]	Soft lithography [62,67]. 3D printing [67]. CHANNEL SIZE Microchannel network: 10 mm diameter [62] Bottom channels: 1000 µm height, 500 µm width Bottom chamber: 10 mm diameter [67]	Immortalized human keratinocytes (HaCaT) [67] Dermal fibroblasts [67] Immune cells [62]. Human umbilical vein endothelial cells (HUVECs) [59] Human primary microvascular endothelial cells [59]	Pharmacological study for the treatment of acne [67] Study of viral infections [62] Pharmacological study of the skin sensory system [63,66]
Cornea-on-chip	Formation of an epithelial and an endothelial corneal compartment, comprising elements of the extracellular matrix [75] Presence of a biomimetic eyelid to mimic the blinking of eyelashes [76]	Lack of stromal behavior may not help in treating more severe lesions [75]	3D printing and 3D cell patterning technique [76] Photolithography and soft lithography [75] CHANNEL SIZE Channels: 220 µm height Hole chamber: 6 mm [75]	Human corneal epithelial cells (HCEpi) [75] Human corneal endothelial cells (HCEnd) [75] Primary human keratinocytes [76], human corneal epithelial cells, primary human corneal fibroblasts	Study of ocular mechanisms and discovery of new ophthalmological drugs [76] Corneal epithelial wound repair investigation [75]
Airway-on-chip	Presence of mechanical stimulus to mimic breathing and presence of air–liquid interface (ALI) [27,28,84,85,87]. Presence of biosensors [27,28,85,86] Presence of two compartments to mimic the epithelium (subjected to the presence of air–endothelium interface) [84,85,87]	Biomechanical ventilation generation [99] Differentiation process of hAECs in 62 days of culture [129]	Stereolithography [83] Soft lithography [82,84] Manufacturing on glass 3D printing with a microfluidic dispenser [85] Laser micromachining E-beam evaporation [84] CHANNEL SIZE Top/apical channel: 1000 µm width, 1000 µm height [83,87] Bottom/basal channel: 1000 µm width, 200 µm height [83,87] Microfluidic channel: 500 µm width Culture chamber: 6.5 mm width Channel pattern: 800 µm width, 300 µm height [85]	Primary human airway epithelial cells (hAECs) [36] Human lung adenocarcinoma cells (NCI-H1437) [85]	Monitoring the cytotoxicity of drugs used to treat lung cancer [85] Drug testing for the treatment of viral infections or to prevent them (i.e., SARS-CoV-2) [87] Study the effects of silica nanoparticles to model the toxic effects of airborne particles [82]
Gastrointestinal barrier-on chip	Presence of two membrane-separated chambers to simulate the crosstalk between primary gastric cells and antral epithelial cells [106] Presence of a multilayer to recreate the intestinal epithelial endothelium [100,104] Integration of sensors to monitor oxygen concentration for the growth of anaerobic bacteria in the gut [84]	Challenges in gastric organoid formation [106] Ensure anaerobic conditions for the growth of anaerobic bacteria [104]	Photolithography and soft lithography [99,101] Laser ablation [101] CHANNEL SIZE Upper and lower channels: 150 µm height, 1000 µm width [99] Upper channel: 700 µm height Bottom channel:400 µm height Afferent channel: 0.8 mm width, 0.6 mm height Efferent channel: 2 mm width, 0.4 mm height [100] Channel: 110–120 µm height [101]	Primary gastric mesenchymal stromal cells (gMSCs) [106] Epithelial cells derived from human antral organoids (hAOs) [106] Human peripheral blood mononuclear cells (PBMCs) [106] Human umbilical cord vein endothelial cells (HUVECs) [100] Human colorectal carcinoma epithelial cells (Caco-2) [100]	Investigate gastric defense mechanisms and develop drug therapies [106] Simulating fungal infections in the gut to identify new drug therapies [128] Study of interactions between microbiota and gut to better understand gut diseases [104] Performing adenoviral transduction on a chip for the study of inflammatory molecules [105]
Testis-on-chip	Existence of multiple compartments to simulate the blood–testicular interface [107]	Difficulty in creating a multi-organ-on-chip that involves the testicular apparatus and another tissue (e.g., liver) [107]	Stereolithography [107] Photolithography and soft lithography [110,114] CHANNEL SIZE Shallower connecting channel: 500 µm height, 250 µm length, 125 µm width [107] Culture chamber: 800 µm width, 2500 µm length, 200 µm height Perfusion channel: 812.5 µm length, 200 µm height [107] Microfluidic channel: 250 µm height [110] Tissue culture compartments: one of 6.5 mm diameter and another one of 13 mm. Microchannel: 250 µm height, 1000 µm width [114]	Ex vivo tissue culture of seminiferous tubules of prepubertal marmosets [107] Human liver spheroids (HepaRG cells) [114] Primary human liver stellate cells [114] Human testicular organoids [114]	Analysis of the effect of drugs (i.e., chemotherapeutics) and their metabolites at the testicular level [114] Ex vivo tissue studies to understand the effect of hormonal stimulation [107]
Placenta-on-chip	Presence of the trophoblast–endothelium interface across two compartments separated by a membrane [119,127]	Lack of cell lines characterizing the early stage of gestation [95] Difficulty in carrying out studies of drug transport across the placenta during the early months of gestation [95]	Device purchased from AIM Biotech [126] Soft lithography [127] CHANNEL SIZE Upper and lower channels: 1.5 mm width, 1.5 cm length, 400 µm height [127] Microfluidic channel: 0.5 mm width. Gel channel: 1.3 mm width, 0.25 mm height [126]	Human trophoblasts (BeWo) [119,127] Human placental endothelial villous [119] Human umbilical cord vein endothelial cells (HUVECs) [127] Human macrophages (THP-1) [127] Pluripotent stem cells [22]	Study of the effects of a drug on the fetus [119] Investigate bacterial infections at the placental level that can lead to preterm fetal death [127]

## 10. Conclusions

In summary, organ-on-chip (OoC) technology represents a significant advancement in the study of biological barriers, offering a more accurate and ethical alternative to traditional 2D cell cultures and animal models. These microfluidic platforms provide valuable insights into the complex physiology of barriers such as the blood–brain barrier, the skin, the placenta, and the gastrointestinal barrier, among others. By replicating the dynamic and multifaceted nature of these barriers, OoC systems enable more precise disease modeling, drug testing, and personalized medicine.

By incorporating biomechanical forces such as shear stress and cyclic strain, these systems more accurately replicate the in vivo conditions compared to traditional static culture methods. The emergence of organ-on-chip (OoC) technology represents a paradigm shift in biomedical research, offering a highly biomimetic approach to studying biological barriers and their roles in health and disease, including drug therapies. Traditional models, including animal studies and static in vitro cultures, have long been used to investigate barrier function. However, these models face significant limitations, including ethical concerns, species differences, and the inability to fully replicate the human physiological conditions. In contrast, OoC systems integrate human-relevant cell types, dynamic microenvironments, and real-time monitoring capabilities, which makes them a powerful alternative for studying the complexities of biological barriers. The integration of induced pluripotent stem cells (iPSCs) into OoC platforms further enhances their potential, offering patient-specific models that allow for the exploration of individual responses to therapies and the development of more targeted treatments. Biosensors incorporated into these platforms enable the real-time monitoring of barrier integrity and cellular functions, which is very useful for advancing drug discovery and optimizing therapeutic strategies. Additionally, the use of OoC models for studying host–microbiome interactions in the gut and skin highlights their potential to unravel the complex interplay between human tissues and microbial communities, an area of increasing importance in health and disease research. This review highlights the progress made in developing various barrier-on-chip models, including the blood–brain barrier, blood–retinal barrier, skin, cornea, airway, gastrointestinal barrier, testis, and placenta. These models have demonstrated significant potential for advancing our understanding of barrier physiology, disease mechanisms, and therapeutic responses. While OoC systems are already providing valuable data in disease research and drug testing, challenges remain before barrier-on-chip technology can be widely adopted in clinical and pharmaceutical settings. Standardization across different platforms, reproducibility of the results, and scalability for high-throughput applications are key issues that need to be addressed. Moreover, further validation studies are required to benchmark these models against human clinical data to ensure their reliability for regulatory approval and drug development. The complexity of biological systems, the need for more advanced sensor technologies, and the integration of multi-organ models are areas that require further development. Advances in 3D bioprinting and tissue engineering may further enhance the complexity and functionality of barrier-on-chip models, moving toward fully functional, patient-specific organ models for disease modeling and regenerative medicine. Future advancements, such as the incorporation of artificial intelligence for data analysis and the scaling of these platforms for clinical applications, hold great promise. As these technologies continue to evolve, they could revolutionize preclinical research, reduce the reliance on animal testing, and ultimately lead to more effective, personalized treatments. By faithfully replicating human physiology, OoC systems bring us closer to bridging the gap between in vitro research and clinical outcomes, paving the way for more efficient and precise biomedical advancements. Furthermore, the integration of biosensors enables the real-time assessment of barrier integrity, transport processes, and cellular responses to external stimuli. In conclusion, barrier-on-chip technology stands at the forefront of a new era in biomedical research, offering unprecedented insights into barrier function and disease progression, while paving the way for more effective drug development and precision medicine. With continued interdisciplinary collaboration and technological refinement, these models have the potential to revolutionize the study of human biology and therapeutic innovation.

## Figures and Tables

**Table 1:** Comparison of different study models for diseases with high (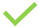), medium (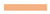), and low (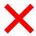) relevance/ability.

	Animal Model 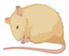	2D Cell Culture 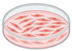	3D Cell Culture 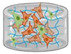	Organ-on-Chip 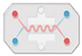	References			
					Animal Model	2D Cell Culture	3D Cell Culture	Organ-on-Chip
Translatability of results	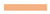	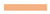	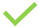	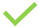	[9,10,11,12,15,24,27,28,29,30,31]	[15,17]	[15,17]	[9,10,11,12,15]
Cell–cell interactions	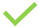	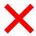	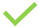	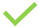	[9,10,11,15]	[15,17]	[15,17]	[9,10,11,15]
Disease model recapitulation	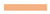	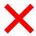	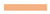	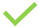	[9,10,11,12,15,31]	[15,17]	[15,17]	[9,10,11,12,15]
Drug discovery	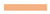	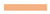	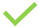	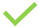	[9,10,11,15]	[15,17]	[15,17]	[9,10,11,15]
Biosensor integration	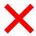	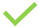	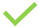	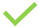	[9,10,11,15]	[15,17]	[15,17]	[9,10,11,15,24,26]
Ethical issues	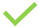	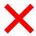	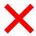	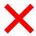	[9,10,11,15,31]	[15,17]	[15,17]	[9,10,11,15]

## Data Availability

No data were used for the research described in the article.

## Abbreviations

OoC	organ-on-chip
hiPSCs	human induced pluripotent stem cells
BBB	blood–brain barrier
BRB	blood–retinal barrier
C6-NCs	coumarin-6 nanocrystals
MEAs	multiple-electrode arrays
SNPs	single-nucleotide polymorphisms
ESCs	embryonic stem cells
iBMECs	induced brain microvascular endothelial cells
ZO-1	zonula occludens-1
CSF	cerebrospinal fluid
TEER	transepithelial/transendothelial electrical resistance
ECs	endothelial cells
COP	cyclo-olefin polymer
EIS	electrical impedance spectroscopy
hCMEC/D3	human brain microvascular endothelial cells
iPS-BMVECs	human brain microvascular endothelium derived from induced pluripotent stem cells
VE-cadherin	vascular endothelial cadherin
iBRB	inner blood retinal barrier
oBRB	outer blood retinal barrier
RPE	retinal pigment epithelial
ILM	inner limiting barrier
GCL	ganglion cell layer
INL	outer nuclear layer
OS	outer segments
AMD	age-related macular degeneration
PDMS	polydimethylsiloxane
HUVECs	human umbilical vein endothelial cells
ARPE-19	retinal pigment epithelium cells
CNV	choroidal neovascularization
HRECs	primary human retinal endothelial cells
SH-SY5Y	human neuroblastoma cell line
DRIE	deep reactive-ion etching
NeuN	neuronal nuclei
MAP2	microtubule-associated protein 2
MVN	microvascular networks
HRMVEC	human retinal microvascular endothelial cells
HRP	primary human retinal microvascular pericytes cells
HRA	primary human retinal astrocytes cells
VEGF	vascular endothelial growth factor
CoCl_2_	cobalt chloride
ALI	air–liquid interface
SoC	skin-on-chip
HSV	herpes simplex virus
TRPV1	transient receptor protein-villanoid-1
IC-SoC	interface-controlled skin-on-chip
P. acnes	Propionibacterium acnes
PET	polyethylene terephthalate
HaCaT	human keratinocytes cells
HCEpi	human corneal epithelial cells
HCEnd	human corneal endothelial cells
EPI-ALI	(air–liquid interface)–(epithelial)
Papp	permeability coefficient
FITC	fluorescein 5(6)-isothiocyanate
MMP-2	matrix metalloproteinase-2
CK-3/12	cytokeratin 3/12
DAPI	4′,6-diamidino-2-phenylindole
COPD	chronic obstructive pulmonary disease
TNF-α	tumor necrosis factor α
ICAM-1	intercellular adhesion molecule 1
ITO	indium tin oxide
NCI-H1437	human lung adenocarcinoma cells
CI	cellular index
SARS-CoV-2	severe acute respiratory syndrome coronavirus
CK-5	cytokeratin 5
GI	gastrointestinal
IBD	inflammatory bowel disease
LGG	Lactobacillus rhamnosus GG
PECAM-1	platelet endothelial cell adhesion molecule 1
Caco-2	human colorectal adenocarcinoma cells
CCL20	C-C motif ligand 2
hAOs	human antral organoids
gMSCs	primary gastric mesenchymal stromal cells
PBMCs	human peripheral blood mononuclear cells
NF-KB	nuclear factor KB
BTB	blood–testis barrier
CYP450	cytochrome P450
HepaRG	human liver spheroids
HPVECs	placental vascular endothelial cells
BeWo	human placental choriocarcinoma cells
FKBPL	binding protein FK506
Gal-3	galectin 3
THP-1	human macrophages
IL-1 α/β/8	interleukin 1 α/β/8
